# An international longitudinal registry of patients with atrial fibrillation at risk of stroke (GARFIELD): the UK protocol

**DOI:** 10.1186/1471-2261-13-31

**Published:** 2013-04-23

**Authors:** Patricia N Apenteng, Ellen T Murray, Roger Holder, F D Richard Hobbs, David A Fitzmaurice

**Affiliations:** 1 Primary Care Clinical Sciences, School of Health and Population Sciences, University of Birmingham, Edgbaston, Birmingham, B15 2TT, UK; 2 Primary Care Health Sciences, University of Oxford, 23-38 Hythe Bridge Street, Oxford, OX1 2ET, UK

**Keywords:** Anticoagulation, Atrial fibrillation, Registry, Stroke, Vitamin K antagonists

## Abstract

**Background:**

Atrial fibrillation (AF) is an independent risk factor for stroke and a significant predictor of mortality. Evidence-based guidelines for stroke prevention in AF recommend antithrombotic therapy corresponding to the risk of stroke. In practice, many patients with AF do not receive the appropriate antithrombotic therapy and are left either unprotected or inadequately protected against stroke. The purpose of the Global Anticoagulant Registry in the FIELD (GARFIELD) is to determine the real-life management and outcomes of patients newly diagnosed with non-valvular AF.

**Methods/design:**

GARFIELD is an observational, international registry of newly diagnosed AF patients with at least one additional investigator-defined risk factor for stroke. The aim is to enrol 55,000 patients at more than 1000 centres in 50 countries worldwide. Enrolment will take place in five independent, sequential, prospective cohorts; the first cohort includes a retrospective validation cohort. Each cohort will be followed up for 2 years. The UK stands to be a significant contributor to GARFIELD, aiming to enrol 4,582 patients, and reflecting the care environment in which patients with AF are managed. The UK protocol will also focus on better understanding the validity of the two main stroke risk scores (CHADS_2_ and CHA_2_DS_2_VAS_C_) and the HAS-BLED bleeding risk score, in the context of a diverse patient population.

**Discussion:**

The GARFIELD registry will describe how therapeutic strategies, patient care, and clinical outcomes evolve over time. This study will provide UK-specific comprehensive data that will allow a range of evaluations both at a national level and in relation to global data and contribute to a better understanding of AF management in the UK.

**Trial registration:**

ClinicalTrial.gov: NCT01090362

## Background

Atrial fibrillation (AF) is the most common clinically significant arrhythmia in the adult population; it is an independent risk factor for stroke and mortality. People with AF have a fivefold increased risk of stroke and a twofold increased risk of death [1]. Prevalence of AF increases throughout life, affecting less than 1% of individuals under 60 years, approximately 4% of individuals over 60 years, and up to 10% of over those aged 80 years [2,3].

The estimated diagnosed prevalence of AF in the UK is around 1.4% [4,5], and more than 46,000 new cases of AF are diagnosed every year [6]. About 15% of all strokes are caused by AF, and 12,500 strokes each year in England are thought to be directly attributable to AF [7]. Furthermore, AF-related strokes are more serious: they are more likely to be fatal than strokes in patients without this arrhythmia; among patients who survive, these strokes cause more disability with less likelihood of independent recovery [8]. For example, findings from the Framingham study indicate that mortality is increased 1.84-fold in strokes in people with AF compared to those in sinus rhythm, and recurrence is more frequent [9]. The Copenhagen stroke study found that patients with AF require longer hospital stays (50 days versus 40 days, P < 0.001) and a lower discharge rate to their own homes (odds ratio 1.7; 95% confidence interval [CI] 0.44 to 0.85) with poorer neurological and functional outcomes [8]. Further, data from the European community stroke project show that AF increased by 50% the probability of remaining disabled (odds ratio 1.43; 95% CI 1.13 to 1.80) or handicapped (odds ratio 1.51; 95% CI 1.13 to 2.02) [10].

Management of AF requires either a rate-control strategy to slow the ventricular rate or a rhythm-control strategy in an attempt to maintain sinus rhythm. Regardless of whether the rate-control or the rhythm-control strategy is pursued, antithrombotic therapy for prevention of stroke and thromboembolism is a fundamental management tool.

Oral anticoagulants are effective in the reduction of stroke and thrombolytic events among patients with AF. Vitamin K antagonists (VKAs) are the most widely used anticoagulants and adjusted-dose warfarin has been shown to reduce the risk of stroke by approximately 60% in patients with AF [9]. However, in practice the use of VKAs is not universal [10]. As a result, only about one-half of the patients who should receive antithrombotic therapy to prevent thromboembolic stroke actually receive it [11].

Risk stratification is important when considering anticoagulation, as the risk of stroke in AF patients is dependent on clinical predictors [12]. A recent stroke risk stratification scheme, CHA_2_DS_2_-VASc (Cardiac failure, Hypertension, Age ≥75 [Doubled], Diabetes, Stroke [Doubled] – Vascular disease, Age 65–74 and Sex category [Female]), has been proposed as an alternative to CHADS_2_[13]. CHA_2_DS_2_-VASc adds further variables to CHADS_2_ – age 65–74, female sex, and vascular disease, and thromboembolism in addition to stroke/ transient ischaemic attack (TIA).

Anticoagulant therapy carries a risk of bleeding, and major bleeding such as intracranial bleeds can be catastrophic. Bleeding risk-stratification schemes assess the risk of major bleeding for patients on anticoagulation to help determine the risk–benefit balance in AF. A novel bleeding risk score – HAS-BLED (Hypertension, Abnormal renal/liver function [1 point each], Stroke, Bleeding history or predisposition, Labile international normalised ratio [INR], Elderly [>65], Drugs/alcohol concomitantly [1 point each]) [14] – is gaining recognition internationally [12] and in the UK, and could potentially improve assessment of bleeding risk in patients with AF.

**Figure 1:**
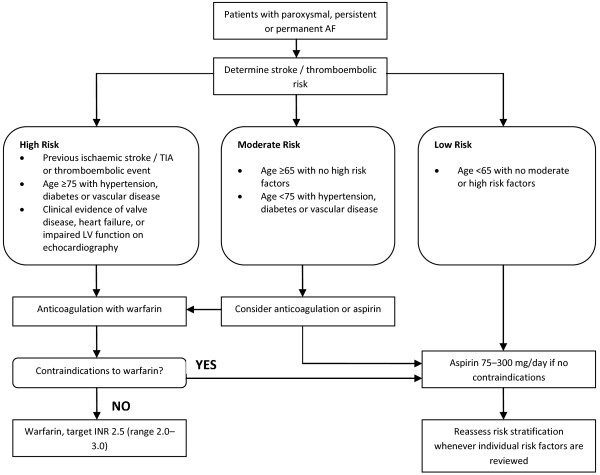
**NICE stroke risk stratification and thromboprophylaxis algorithm [**[15]**].** Reproduced with permission.

In 2006 the National Institute for Health and Clinical Excellence (NICE) published guidelines for the management of AF, with priorities on identification and diagnosis of AF, treatment of AF, and provision of antithrombotic therapy [15]. One of the key recommendations of the guidelines is a formal assessment of the risk of thromboembolism using a stroke risk stratification and thromboprophylaxis algorithm (Figure 1). The guideline proposes routine anticoagulation with warfarin for patients at high risk of stroke, and aspirin for those at low risk of stroke.

The prevalence of AF in the UK is increasing, probably due to the ageing population and improved survival from conditions predisposing to AF, including, for example, myocardial infarction. A large population-based study of the epidemiology and treatment of AF in the UK found prevalence of diagnosed AF rose steadily (0.84% in men in 1994 compared with 1.49% in 2003, compared with 0.83% and 1.29%, respectively, in women) [4]. The number and proportion of AF patients in the UK prescribed antithrombotic therapy has progressively increased over time [4,16]. An analysis of national data from 1994 to 2003 found under one-half of all AF patients received any antithrombotic treatment in 1994 but around 80% received some sort of stroke prevention in 2003 [4]. Also, treatment of AF with oral anticoagulants more than doubled from 1994 to 2003 in men (25% to 53%) and has increased significantly women (32% to 40%) [4]. However, the use of anticoagulants remains inappropriate [4,17] and the NICE 2006 costing report estimated that 46% of patients who should be on warfarin are not receiving it [18]. There is also evidence to suggest underuse of anticoagulation in the elderly; for example, in one study elderly patients (age >85 years) were less likely to initiate warfarin (relative rate 0.16, 95% CI 0.15 to 0.18) and more likely to start aspirin (relative rate 1.66, 95% CI 1.47 to 1.88) compared with patients aged 40–64 years [17].

Much of the UK evidence is based on retrospective cross-sectional studies and was derived from prevalence data. As such, there is limited evidence on persistence of treatment with antithrombotic therapy and it has been indicated that only 60% of patients prescribed warfarin continue for at least 2 years [17]. Also, much of the available evidence relates to AF management prior to the publication of the NICE guidelines in 2006. It is not clear how well clinicians adhere to these guidelines and what impact this has had. There is an absence of contemporary longitudinal data on the clinical management of AF in the UK, including the key therapeutic area of antithrombotic therapy, persistence of therapy, and related clinical outcomes.

### Importance of GARFIELD UK

The Global Anticoagulant Registry in the FIELD (GARFIELD) is an observational, international, longitudinal registry of patients newly diagnosed with AF at risk of stroke, and aims to determine real-life treatment patterns and clinical outcomes. The global study aims to recruit 55,000 patients in five sequential cohorts of 10,000 patients each, alongside a validation cohort of 5000 patients. The methods for the global study have been published [19]. The UK is the only country undertaking GARFIELD to have its own protocol; the UK protocol was developed from the global protocol and adapted to the UK context to maximise the value of GARFIELD to the UK. It therefore has a slightly different design and includes important and original specific research questions relevant to the UK population. Tailoring the protocol to the UK allowed it to be adopted by the Primary Care Research Network portfolio of research. A number of publications will emanate from the UK-specific data over the course of the study to provide real-life contemporary evidence. As such, this paper is an important point of reference for the UK study. Principally, the UK study will review the management of AF in the UK and evaluate clinical practice against guideline recommendations.

## Methods/design

### Study design

**Figure 2:**
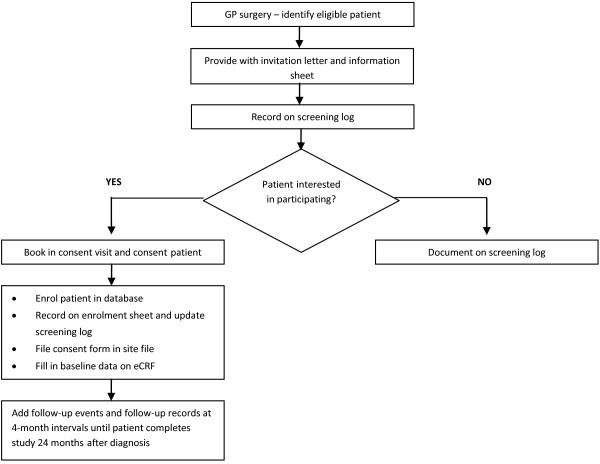
Summary of UK study design.

GARFIELD in the UK is primary care based and aims to recruit 4,582 patients at more than 100 sites across the UK. Enrolment will take place in five independent sequential cohorts, parallel to the global study. Similar to the global study, Cohort 1 will include a retrospective validation cohort of patients diagnosed with AF between 6 months and 24 months previously. Data will be extracted through a case notes review at baseline, and at every 4 months until 24 months after diagnosis. The data will be collected using an electronic case report form (eCRF). A summary of the UK study design is provided in Figure 2.

### Study aims

The key aims of the GARFIELD registry are to determine the real-life treatment patterns and clinical outcomes of newly diagnosed patients with non-valvular AF with at least one additional risk factor for stroke.

In accordance with the global objectives, the study will assess the rate of stroke and systemic embolisation, and assess the outcome of these events with specific reference to:

• The incidence and characteristics of bleeding complications (e.g. location and severity, classified as major, clinically relevant non-major, and minor);

• Therapy persistence, including discontinuation, interruption, and changes of therapy regimen;

• For patients on VKAs, fluctuations in the INR over time.

The UK protocol has additional objectives that will inform the management on AF. GARFIELD in the UK seeks to evaluate the performance of the novel stroke risk score CHA_2_DS_2_-VASc in comparison with CHADS_2_ in predicting stroke risk in the UK study population. Likewise it will evaluate the effectiveness of the bleeding risk score HAS-BLED in predicting bleeding risk within the UK study population. In addition, the study will determine the clinician and patient factors associated with the decision to anticoagulate patients. Another unique objective of the UK study is to determine any variations in levels of anticoagulation associated with ethnicity. Furthermore, the study will determine where patients are principally diagnosed with AF and assess the role of primary care in the management of AF in the UK.

### Study setting

In the UK, all healthcare delivery is centred on the general practitioner (GP), with referrals for specialists and for routine admission to hospital organized at the GP level. As a result of the National Health Service structure, GPs maintain in their surgeries a complete medical history of their patients. Recruiting from the general practices will therefore capture all patients diagnosed with AF regardless of their care settings, and in the UK, these include hospital departments (cardiology) and emergency settings. The UK is therefore recruiting solely from the primary care setting; nevertheless, we expect to achieve a representative sample of patients with AF being cared for in the UK, comparable to the sample recruited in the global study.

Investigator sites (GP practices) will be representative of the UK, and will include sites in England, Wales, Scotland, and Northern Ireland, with the aim of achieving a sample representative of the geographical distribution of the UK population. Practices will be recruited and trained in collaboration with national research networks. The Primary Care Research Network (PCRN) England provides a world-class infrastructure to conduct clinical research in primary care settings in the NHS by supporting and facilitating recruitment and set up of sites. PCRN England is delivered through eight local research networks that cover the whole of England. Similar networks operate in Scotland (Scottish PCRN), Northern Ireland (Northern Ireland Clinical Research Network), and Wales (National Institute for Social Care and Health Research). Expressions of interest will be sent to practices by the research network for each region, and sites will be selected from the responses received.

### Registry population

The details of the registry population are given in full in the global GARFIELD methods paper [19].

#### Prospective cohorts

The eligibility criteria for the prospective cohorts are: patients aged 18 years or older with a diagnosis of non-valvular AF within the past 6 weeks and at least one additional risk factor for stroke [19].

#### Retrospective cohort

The eligibility criteria for the retrospective cohort are: patients with a diagnosis of non-valvular AF within the 6–24 months before enrolment, and at least one additional risk factor for stroke [19].

### Patient recruitment

Each participating GP will identify eligible patients using a search of the computerised clinical record and will invite them by standard letter to be enrolled in the GARFIELD registry. GPs will also opportunistically inform patients in the practice and give them a participant invitation letter and information sheet.

For the retrospective cohort, a practice computer search for all patients with a current diagnosis of AF (between 6 months and 2 years prior to inclusion) will be undertaken. Once identified, patients will be assessed according to the inclusion/exclusion criteria and eligible patients invited to participate.

For the prospective cohort, a computer search will be undertaken at least once a month at each practice to identify newly diagnosed patients with non-valvular AF. Once identified, the patient will be assessed according to the inclusion/exclusion criteria. Eligible patients will be sent a participant invitation letter and information sheet and asked to contact the practice if they are interested in participating. A screening log of all patients invited to participate in the registry will be maintained at each site. A consent visit is arranged for interested patients, after which they are enrolled in the registry and baseline data are completed.

### Collection of baseline and follow-up data

Data collected at baseline include: demographics (e.g. ethnicity, sex, date of birth); body mass index; vital signs at diagnosis; AF symptoms; type of AF (new, paroxysmal, persistent, permanent); method and site of diagnosis; treatment strategy initiated at diagnosis; antithrombotic therapy; treatment decision (patient and physician factors); and medical history (cardiovascular, medical, bleeding).

Follow-up data include clinical events (stroke/TIA, peripheral embolism, acute coronary syndrome) and outcome of event; AF-related medical consultation and/or hospitalisation and outcome; AF treatment change; change in antithrombotic therapy (discontinuation, duration on therapy, reasons for discontinuation); bleeding events (classified as major, clinical relevant non-major, and minor); bleeding location of treatment (e.g. Accident and Emergency, GP practice); outcome of bleeding (recovered, permanently disabled, fatal); bleeding healthcare utilisation (hospitalisation, Accident and Emergency, physician, etc.); medical history update; mortality, including sudden cardiac death and non-cardiovascular death; and INR records in relation to therapeutic range, and location of INR monitoring.

### Clinical outcomes and data quality

The study outcomes comprise clinical events (stroke, TIA, systemic and pulmonary embolism, myocardial infarction), bleeding events, therapy persistence, hospital visits and INR monitoring, and are listed in below:

• Cerebrovascular events defined as stroke including:

– Primary ischaemic stroke

– Primary intracerebral haemorrhage

– Secondary haemorrhagic ischaemic stroke

• TIAs

• Systemic embolism

• Pulmonary embolism

• Mortality

• Acute coronary syndromes including:

– Unstable angina

– ST-elevation myocardial infarction

– Non-ST-elevation myocardial infarction

• Bleeding events including:

– Frequency

– Location

– Severity (classified as major [clinically overt bleeding associated with a fall in haemoglobin of ≥2 g/dl OR a transfusion of ≥2 packed red blood cells or whole blood OR a critical site: intracranial, intraspinal, intraocular, pericardial, intra-articular, intramuscular with compartment syndrome, retroperitoneal OR a fatal outcome], clinical relevant non-major and minor)

• Therapy persistence, including:

– Rate of discontinuation

– Duration of time on therapy

– Reasons for discontinuation

– Duration and cause of treatment interruption or suspension

• Analysis of events listed with regard to hospitalisation and outcomes

• Any other hospital visits (inpatient, outpatient, emergency department)

• Major adverse cardiac events

• For patients treated with VKA:

– Frequency and timing of monitoring required in maintaining therapeutic anticoagulation

– INR recordings in relation to therapeutic range

– Location of INR monitoring and medical consultations due to INR testing

– Use of bridging anticoagulation necessitated by VKA interruption

– Outcomes in relation to INR fluctuation.

Source data verification will be undertaken in 20% of all cases to verify adherence to the protocol and assess the level and accuracy of data recording.

### Funding

The GARFIELD Registry is sponsored by the Thrombosis Research Institute, London, UK. Funding of the registry is provided through an educational research grant from Bayer Pharma AG, Berlin, Germany.

### Sample size and data analysis

The total projected sample size for the UK is 4,582, comprising 417 retrospective patients for Cohort 1 and 833 prospective patients for Cohorts 1 to 5. With these projected sample sizes the precision of estimated incidence of stroke is set out in Table 1 for different levels of incidence.

**Table 1:** Precision of estimated incidence of stroke for different levels of incidence, and confidence intervals on effect sizes for quantitative measures

	**Width of 95% confidence interval**		
**Expected incidence of stroke**	**Sample size of retrospective patients: 417**	**Cohort size of prospective patients: 833**	**Total sample size of 4,582**
2.5%	±1.5%	±1.1%	±0.5%
5%	±2.1%	±1.5%	±0.6%
10%	±2.9%	±2.0%	±0.9%
20%	±3.8%	±2.7%	±1.2%
30%	±4.4%	±3.1%	±1.3%
40%	±4.7%	±3.3%	±1.4%
50%	±4.8%	±3.4%	±1.4%
Width of 95% confidence interval			
Sample size of retrospective patients: 417		Cohort size of prospective patients: 833	Total sample size of 4,582
0.14		0.10	0.04

An interim analysis will be done at the UK level for the baseline data in each cohort and after all patients in each of the first four cohorts have completed the study. For a full analysis, baseline data, follow-up data, and study endpoint data will be summarised overall and by treatment groups, cohort, and region. Summaries of categorical data will be presented as frequency counts, percentages, and 95% confidence intervals. Continuous data will be presented as means (standard deviations), medians (with 95% confidence intervals), interquartile ranges, minimum, maximum, and number of patients.

Comparison of follow-up and outcome data between treatment groups, cohorts, and regions will be made using linear (for continuous outcomes) or non-linear (for categorical outcomes) mixed modelling with practice included as a random effect. Association between outcome variables and baseline data will be explored using the same method. For continuous data, normality of residuals will be tested using the Kolmogorov–Smirnov test and transformation or bootstrapping will be implemented where required. Time-to-event analysis will use Kaplan–Meier and Cox regression analyses to summarise and explore the association with baseline and other pertinent data. Comparison of CHADS_2_, CHA_2_DS_2_VASc, and HAS-BLED risk measures will be compared on the basis of receiver-operating characteristic curve analyses. The baseline characteristics of the patients – who have been classified as at risk of stroke according to physician-perceived risk factors or combinations of factors – will be reported.

## Discussion

The development of this large, ongoing registry allows the opportunity to answer several research questions that have not previously been investigated within a non-randomised, non-selected population. These questions will pertain to:

• Clinical risks within a non-selected population of newly diagnosed patients with AF, compared with data from randomised trials in which prevalent, stable VKA users were preferably enrolled [20];

• Risks and benefits associated with oral anticoagulation;

• Quality of INR control in everyday clinical practice;

• Persisting barriers to prescribing oral anticoagulation;

• The economic burden of AF;

• The main diagnostic pathways, including the real-life identification and management of patients at various levels of risk for ischaemic stroke.

GARFIELD UK data will provide a comprehensive description of AF management and insights into the rationale for decisions relating to anticoagulation. The findings will establish how well the NICE guidelines have been implemented in the UK. Whilst NICE guidelines are not mandatory, they are evidence-based and internationally recognised to reflect best practice. Further, GARFIELD will inform on the effectiveness of the NICE treatment guidelines and allow an evaluation of such guidelines and patient outcomes.

The global data will provide comparable information within which to consider national data and models of best practice, and the significance of the context in interpreting findings. The range of data will also provide evaluation of any inequalities in the UK in terms of AF diagnosis, management, and possibly clinical outcomes. The study will provide the opportunity to identify differences in management and outcomes across care settings, and will offer clarity relating to the effectiveness of INR control within the various test settings in the UK, as well as the effectiveness of the recent stroke (CHA_2_DS_2_VASc) and bleeding (HAS-BLED) risk scores.

The study will provide real-world prospective data that will allow an evaluation of clinical practices and related outcomes in the VKA-only era, but will also report on outcomes relating to any novel anticoagulants or new therapies licensed for use in the UK during the duration of the study.

## Appendix

UK GARFIELD Investigators

David A Fitzmaurice at the University of Birmingham and the UK Clinical Research Network (Primary Care). Will Murdoch, Naresh Chaunan, Daryl Goodwin, Richard McManus, Ramila Patel, Philip Saunders, Bennett Wong, Richard Evans, Philip Saunders, Janet Leese, Prem Jhittay, Andrew Ross, Manjit Kainth, Kevin Douglas, Gill Pickavance, Joanna McDonnell, Andrea Williams, Trevor Gooding, Helga Wagner, Geert Van Zon, Kevin Jones, Shoeb Suryani, Matt Thomas, Emily Watson, Arun Singal, William Wilcock, Subharsi Sircar, John Cairns, Drew Gilliland, Roman Bilas, Elizabeth Strieder, Peter Hutchinson, Anne Wakeman, Michael Stokes, Alistair Howitt, Bhaskhar Vishwanathan, Nigel Bird, Dominic Gray, Paul Evans, Matt Clark, John Bisatt, Jennifer Litchfield, Elizabeth Fisher, Tim Fooks, Richard Kelsall, Neil Paul, Elizabeth Alborough, Michael Aziz, Cobarsanellore Ramesh, Peter Wilson, Simon Franklin, Sue Fairhead, Julian Thompson, Vivien Joseph, Gary Taylor, Huw Charles, Dawn Tragen, Wendy Molefi-Youri, David Seamark, Carolyn Paul, Mark Richardson, Angus Jefferies, Helen Sharp, Hywel Jones, Claire Giles, Michael Page, Olaleye Oginni, Jehad Aldegather, Simon Wetherell, William Lumb, Phil Evans, Frances Scouller, Neil Macey, Stephen Rogers, Yvette Stipp, Richard West, Stephen Thurston, Paul Wadeson, John Matthews, Preeti Pandya, Andrew Gallagher, Raj Priyadharshan, Jayne Oliver, Tammy Railton, Emyr Davies, Steven Sayers, Claire Hutton, Nick Walls, Richard Thompson, Bijoy Sinha, Keith Butter, Susan Barrow, Helen Little, David Russell, Jason Davies, Ikram Haq, Paul Ainsworth, Claire Jones, Phil Weeks, Jane Eden, David Kernick, Janet Glencross, Alison MacLeod, Karen Poland, Connor Mulholland, Alison Warke, Paul Conn, Gerry Burns, Richard Smith, Simon Lowe, Rakee Kamath.

## Abbreviations

AF: Atrial fibrillation; CHA2DS2-VASc: Cardiac failure, Hypertension, Age ≥75 [Doubled], Diabetes, Stroke [Doubled] – Vascular disease, Age 65–74 and Sex category [Female]); CHA2DS2-VASc: Cardiac failure, Hypertension, Age ≥75 [Doubled], Diabetes, Stroke [Doubled] – Vascular disease, Age 65–74 and Sex category [Female]; CHADS2: Cardiac failure, Hypertension, Age, Diabetes, Stroke (Doubled); GARFIELD: Global Anticoagulant Registry in the FIELD; GP: General practitioner; HAS-BLED: Hypertension, Abnormal renal/liver function (1 point each), Stroke, Bleeding history or predisposition, Labile INR, Elderly (>65), Drugs/alcohol concomitantly (1 point each); INR: International normalised ratio; TIA: Transient ischaemic attack.

## Competing interests

PA, ETM, and RH declare that they have no competing interests.

DAF has received honoraria from Bayer, Pfizer, Leo Laboratories, Roche Diagnostics.

FDRH has no direct competing interests but has received sponsorship or consulted for companies with an interest in anticoagulation including Bayer, Boehringer Ingelheim and Pfizer.

## Authors’ contributions

PA contributed to the development of the study protocol, drafted the manuscript and is managing the UK cohorts. ETM is a co-investigator for the UK and is involved in supporting the management of the UK cohorts. ETM contributed to drafting of the study protocol and provided comments on the draft manuscript. RH is a statistician involved in data analysis of the UK cohorts, performed sample size calculations and contributed to drafting of the manuscript. FDRH is a co-investigator for the UK and contributed to the drafting of the UK protocol. FDRH also provided comments on the draft manuscript. DAF is a Steering Committee member and the UK National Coordinator for the GARFIELD Registry. DAF participated in the conception of the project and the study design, drafting of the study protocol, and provided comments on the draft manuscript. All authors have read all of the different versions and approved the final version of this manuscript.

## Pre-publication history

The pre-publication history for this paper can be accessed here:

http://www.biomedcentral.com/1471-2261/13/31/prepub
